# Diffusion-relaxation scattered MR signal representation in a multi-parametric sequence

**DOI:** 10.1016/j.mri.2022.05.007

**Published:** 2022-05-11

**Authors:** Fabian Bogusz, Tomasz Pieciak, Maryam Afzali, Marco Pizzolato

**Affiliations:** a AGH University of Science and Technology, Kraków, Poland; b LPI, ETSI Telecomunicación, Universidad de Valladolid, Valladolid, Spain; c Leeds Institute of Cardiovascular and Metabolic Medicine (LICAMM), Leeds, United Kingdom; d Cardiff University Brain Research Imaging Centre (CUBRIC), Cardiff University, Cardiff, United Kingdom; e Department of applied mathematics and computer science, Technical University of Denmark, Kongens Lyngby, Denmark; f Signal Processing Lab (LTS5), École Polytechnique Fédérale de Lausanne, Lausanne, Switzerland

**Keywords:** diffusion MRI, diffusion-relaxation, multi-parametric sequence, brain, microstructure

## Abstract

This work focuses on obtaining a more general diffusion magnetic resonance imaging (MRI) signal representation that accounts for a longitudinal T_1_ and transverse ${\text{T}}_{1}^{⋆}$ relaxations while at the same time integrating directional diffusion in the context of scattered multi-parametric acquisitions, where only a few diffusion gradient directions and b-values are available for each pair of echo and inversion times. The method is based on the three-dimensional simple harmonic oscillator-based reconstruction and estimation (SHORE) representation of the diffusion signal, which enables the estimation of the orientation distribution function and the retrieval of various quantitative indices such as the generalized fractional anisotropy or the return-to-the-origin probability while simultaneously resolving for T_1_ and ${\text{T}}_{1}^{⋆}$ relaxation times. Our technique, the Relax-SHORE, has been tested on both *in silico* and *in vivo* diffusion-relaxation scattered MR data. The results show that Relax-SHORE is accurate in the context of scattered acquisitions while guaranteeing flexibility in the diffusion signal representation from multi-parametric sequences.

## 1 Introduction

Diffusion and relaxation are two complementary mechanisms that allow the *in vivo* examination of the brain tissue microstructure in a non-invasive way with magnetic resonance imaging (MRI). The former provides information about the diffusive motion of water molecules on a microscopic level and is sensitive to the geometry of the tissue’s microstructure. The latter is summarized by the longitudinal and transverse relaxation times, T_1_ and ${\text{T}}_{2}/{\text{T}}_{2}^{⋆}$, which are a result of the physicochemical interactions of water molecules with their surroundings.

The diffusion process can be represented in many different ways depending on the chosen acquisition scheme. These include the apparent diffusion coefficient (ADC), which reflects the mean diffusion inside a voxel (Le Bihan et al., 1986), diffusion tensor imaging (DTI) (Basser et al., 1994), high angular resolution diffusion imaging (HARDI) (Tuch, 2004), diffusion kurtosis imaging (DKI) (Jensen et al., 2005), and the q-space methods designed to retrieve the apparent ensemble-average propagator (EAP) from densely sampled data (Wedeen et al., 2005; Wu and Alexander, 2007) eventually collected on multiple “shells” (Özarslan et al., 2013b).

The linear expansion of the diffusion signal in basis functions such as simple harmonic oscillator based reconstruction and estimation (SHORE) (Özarslan et al., 2009) has revealed useful in research and clinical scenarios. Once the basis coefficients are known it is possible to analytically approximate both the diffusion signal and the apparent EAP. Knowledge of this enables the calculation of various quantitative indices that are related, through the diffusion process, to the geometry of the brain’s tissue microstructure. These are, for instance, the return-to-origin probability (RTOP), return-to-axis probability (RTAP), and return-to-plane probability (RTPP) which are related, in the context of pore theory (Callaghan et al., 1992), to the pore’s mean volume, cross-sectional area, and length respectively (Özarslan et al., 2013b). These quantitative indices are of interest to research and clinicians as they provide insights into tissue heterogeneity (Brusini et al., 2018), alterations in ischemic stroke (Brusini et al., 2016; Boscolo Galazzo et al., 2018), cognitive impairment detection (Pitteri et al., 2021), aging and dementia (Moody et al., 2021), and multiple sclerosis affecting gray matter areas (Brusini et al., 2021).

The complexity of biological material makes the precise assessment of tissue properties challenging (Beaulieu and Allen, 1994; Jones et al., 2013; Afzali et al., 2021). To gather more information about the tissue, multi-parametric acquisitions have been used to simultaneously characterize diffusion and relaxation phenomena. In these acquisitions (Hutter et al., 2018; Leppert et al., 2021), the inversion time (TI) and/or echo time (TE) are changed while applying a diffusion weighting, generally described by a b-value. By varying TI it is possible to estimate the longitudinal relaxation time, T_1_, while the use of multiple TEs allows estimating the transverse relaxation time, T_2_, or the effective one ${\text{T}}_{2}^{⋆}$.

Multi-parametric acquisitions typically include an extensive collection of measurements at different b-values, TIs, or TEs, which entails long acquisition times that are unpractical in a typical clinical routine. To cope with this, multi-parametric strategies like slice-shuffled inversion- recovery (Leppert et al., 2021), MESMERISED (an acronym for Multiplexed Echo Shifted Multiband Excited and Recalled Imaging of STEAM Encoded Diffusion) (Fritz et al., 2021) or ZEBRA (acronym for Z-location shuffling, multiple Echos and B-interleaving for Relaxometry-diffusion Acquisitions) (Hutter et al., 2018) have been developed to sample the acquisition parameter space determined by echo time, inversion time, and b-values in a scattered way. In particular, ZEBRA consists of a simultaneous multislice interleaved acquisition strategy to acquire MRI volumes with unique combinations of TE, TI, b-value, and gradient direction where it is not possible to find any two volumes that share the same TE and TI for a distinct b-value and gradient direction.

To simultaneously analyze the diffusion and relaxation processes, in general, three main approaches are employed, namely continuum modelling, cumulant expansion of the signal, and biophysical modelling (Slator et al., 2021). Continuum models provide information about the diffusion-relaxation correlation spectra. However, many measurements are required due to the ill-posedness of the inverse Laplace transform estimation procedure. The correlation spectra alternatively can be obtained by combining similar neighbouring voxels spectra (Kim et al., 2017) or assuming that spectra along one dimension are the result of marginalization of higher-dimensional spectra (Benjamini and Basser, 2016). Approximation of the diffusion-relaxation signal by its cumulant expansion is another possibility that enables computation of the joint diffusion-relaxation moments (Ning et al., 2019). Lastly, the diffusion-relaxation signal can be characterized with biophysical models that allow distinguishing distinct compartments that contribute to the signal (Panagiotaki et al., 2012). More recently, (Martin et al., 2021) proposed a more general formulation of the multidimensional problem, which includes the use of diffusion encodings based on free waveforms. In this work, we do not follow any of the three approaches mentioned above as we assume the single compartment signal model. We focus mainly on modelling the data in the context of scattered acquisitions, which is not a trivial task *per se*, rather than representing the whole multidimensional spectrum.

Although scattered acquisitions are more efficient from a sampling perspective, the calculation of quantitative diffusion indices, such as the RTOP, requires a continuous representation of the diffusion signal across b-values and directions (Özarslan et al., 2013b; Fick et al., 2016). Indeed, indices like RTOP, RTPP, and RTAP are integral measures of the signal that ideally require the knowledge of the diffusion signal continuously and towards infinite b-values. Therefore, since for each combination of TI and TE only a few diffusion gradient encodings (b-values and directions) are available, scattered acquisitions pose relevant challenges for the estimation of these continuous representations, such as the SHORE.

We propose an estimation strategy for continuously representing the signal comprising relaxation and diffusion decays that is robust for use in the context of scattered acquisitions strategies. We base the representation of the diffusion directional decay on SHORE to guarantee the fidelity of the reconstructed signal on a wide range of b-values and to retain the directional information of the diffusion process. In particular, the proposed estimation strategy consists of alternately repeating two estimation procedures: one for relaxation, based on a nonlinear optimization procedure, and one for the diffusion part of the signal based on linear regression. We show that this strategy allows us to robustly estimate the microstructural diffusion indices and the relaxation times from scattered data such as that collected for the Multidimensional Diffusion (MUDI) challenge (Pizzolato et al., 2020) based on ZE-BRA (Hutter et al., 2018).

## 2 Theory

### 2.1 Non-directional diffusion-relaxation scattered MR signal representation

Considering a simple single-compartment model the signal coming from a multi-parametric sequence can be represented as

(1) $$\begin{array}{l} S(b,\theta )=\text{PD}(1-\text{IE}\exp(-\frac{TI}{{\text{T}}_{1}})+\exp(-\frac{TR}{{\text{T}}_{1}}))\\ \times \exp(-\frac{TE}{{\text{T}}_{2}^{⋆}})\exp(-bADC), \end{array}$$

where *S*(*b*, ***θ***) is the signal parametrized by the sequence variables ***θ*** = (*TE, TI, TR*) namely, echo time *TE*, inversion time *TI* and repetition time *TR, b* is the diffusion weighting, ADC is the apparent diffusion coefficient, PD is the proton density, and IE is the inversion efficiency. Assuming a full inversion and *TR* ≫ *T*_1_, and inversion efficiency to be IE = 2 we can simplify Eq. (1) as follows

(2) S(b,θ)=R(ρ∣θ)exp(−bADC),

where *R*(***ρ***|***θ***) is the relaxation part defined as

(3) $$R(\rho |\theta )=\text{PD}\left(1-2\exp\left(-\frac{TI}{{\text{T}}_{1}}\right)\right)\exp\left(-\frac{TE}{{\text{T}}_{2}^{⋆}}\right)$$

with $\rho =(\text{PD},{\text{T}}_{1},{\text{T}}_{2}^{⋆})$ and the parameter ***θ*** simplified to ***θ*** = (*TE, TI*). The signal representation specified by Eq. (2) is referenced as Relax-ADC as it assumes the ADC to represent the diffusion part in the diffusion-relaxation MR signal. Assuming that the diffusion coefficient is modelled using the gamma distribution one can present the diffusion signal as *S*(*b*) = *S*(0) (1 + (KD*b*)/3)^−3/K^ with K being the kurtosis term and diffusion coefficient D modeled using the gamma distribution (Jensen and Helpern, 2010). A further extension of Relax-ADC thus is the gamma kurtosis diffusion-relaxation MR recently introduced by Bogusz et al. (2020)

(4) $$S(b,\theta )=R(\rho |\theta ){\left(1+\frac{\text{KD}b}{3}\right)}^{-3/\text{K}}.$$

Henceforth, we refer to the representation given in Eq. (4) as to Relax-Kurtosis. However, the representations specified by Eqs. (2) and (4) take no account of the directionality of the diffusion process and are valid mostly for low or/and moderate *b*-values. In the next section, we thus discuss a generalization of these representations that take into account also directionality as well as the non-Gaussian nature of the signal at high b-values.

### 2.2 Directional diffusion-relaxation scattered MR signal representation via a diffusion tensor

To obtain the information about the direction of the diffusion process with a diffusion-relaxation MR acquisition, a signal representation based on DTI has been introduced by De Santis et al. (2016a,b)

(5) $$S(b,\theta ,u)=R(\rho |\theta )\;\exp\;(-bu^{T}Du),$$

where *S*(*b*, ***θ*, u**) is the diffusion-relaxation MR signal acquired in direction **u** being a unit vector and **D** is the diffusion tensor algebraically represented by a symmetric positive-definite matrix of size 3 × 3. This is approach is similar to that presented in Pizzolato et al. (2020). The signal representation in Eq. (5) is referenced as Relax-DTI. This formulation, however, is valid mostly under the low b-value regime and entails all the limitations of DTI Mori and Zhang (2006). Note, however, in the formulations originally presented by De Santis et al. (2016a,b), multiple second-order tensors are used to represent the diffusion part for each fibre separately via a mixture model.

### 2.3 Directional diffusion-relaxation scattered MR signal representation using functional bases

The diffusion MR signal attenuation can be decomposed linearly via basis functions that represent the radial information, i.e. across b-values, and the angular information, i.e. over directions, of the diffusion process. One example of radial profile representation is the socalled one-dimensional SHORE that aggregates orthogonal Hermite polynomials to decompose the diffusion MR signal (Özarslan et al., 2009, 2013a). The three-dimensional SHORE (hereinafter referred to as the 3D-SHORE) extends the one-dimensional version to account for the angular/directional information (Özarslan et al., 2013b; Avram et al., 2016). Although various other functional representations have been proposed so far (e.g., Assemlal et al. (2009); Cheng et al. (2010); Descoteaux et al. (2011); Ning et al. (2015)), 3D-SHORE attracted particular attention in the community as it is characterized by a relatively high re-producibility of quantitative measures (Avram et al., 2016), possibilities in handling outliers (see for example Fick et al. (2016); Koch et al. (2019); Varela-Mattatall et al. (2020) in the case of MAP-MRI basis) and extensions to time-dependent diffusion MRI (Fick et al., 2018; Filipiak et al., 2019). In this paper, however, we use 3D-SHORE as it enables us to represent the directionality of the signal in a fibre crossing scenario more robustly than other solutions (e.g. Fick et al. (2015)) and to avoid the estimation of the anisotropic scaling factor that is required with MAP-MRI.

The 3D-SHORE basis incorporates the product of a radial oscillator and angular spherical harmonics to represent the normalized diffusion MR signal (Özarslan et al., 2009; Cheng, 2012)

(6) $$E(q)=\sum_{l=0,\;\text{even}\;}^{L}\sum_{n=l}^{(L+l)/2}\sum_{m=-l}^{l}a_{nlm}{\text{Φ}}_{nlm}(q),$$

where **q** is the wave vector, *a*_*nlm*_ are the coefficients related to the basis functions Φ_*nlm*_(**q**) given in the form

(7) $${\text{Φ}}_{nlm}(q)=G_{nl}(q)Y_{l}^{m}(u)$$

with radial *G*_*nl*_(*q*) and angular *Y*_l_^*m*^(**u**) parts, defined respectively as

(8) $$\begin{array}{l} G_{nl}(q)\;=\;{\left[\frac{2(n-l)!}{\zeta^{3/2}\Gamma (n+3/2)}\right]}^{1/2}{\left(\frac{q^{2}}{\zeta }\right)}^{l/2}\\ \;\times \exp(-\frac{q^{2}}{2\zeta })L_{n-l}^{l+1/2}(\frac{q^{2}}{\zeta }) \end{array}$$

with Γ(·) being the gamma function, *n* is the radial order, *ζ* is scale parameter estimated from the data, $L_{k}^{\alpha }$ is the associated Laguerre polynomial defined in formula representation (Gradshteyn and Ryzhik, 2014)

$$L_{n}^{\alpha }(x)=\sum_{k=0}^{n}{\left(-1\right)}^{k}\frac{(n+\alpha )!}{(n-k)!(\alpha +k)!k!}x^{k}.$$

and $Y_{l}^{m}(u)$ is the spherical harmonics function of degree *l* and order *m*, **u** = **q**/ ∥**q**∥ is a unit vector, and *q* = ∥**q**∥.

Note that, since diffusion is a real and symmetric process, only even order spherical harmonics components are considered.

Hence, in this work, we represent the diffusion-relaxation signal as:

(9) $$S(q,\theta )=R(\rho |\theta )\sum_{l=0,\;even\;}^{L}\sum_{n=l}^{(L+l)/2}\sum_{m=-l}^{l}a_{nlm}{\text{Φ}}_{nlm}(q).$$

We refer to the representation in (9) as to the Relax-SHORE. This representation describes both relaxation and diffusion while allowing us to account for the directionality of the diffusion process and for the non-Gaussianity that needs to be taken into account at high *b*-values.

### 2.4 Numerical optimization of directional diffusion-relaxation scattered MR signal representations

By assuming homogeneous compartments for relaxation the signal representations defined in Eqs. (5) and (9) can be rewritten by separating the relaxation and diffusion terms as follows:

(10) S(q,θ)=R(ρ∣θ)E(q).

To fit a signal representation given by Eq. (5) or (9), we use a two-step alternating method. This approach includes fitting the relaxation part once fixing the diffusion part to be constant and then fitting the diffusion part leaving the relaxation part untouched. However, in the case of Relax-ADC and Relax-Kurtosis, we estimate all relaxation and diffusion parameters with a single optimization scheme using the mean squared differences between the data and fitted representation that is solved via the Levenberg-Marquardt algorithm. Below, we present the numerical procedures used to fit both directional representations, namely the Relax-DTI and Relax-SHORE.

#### Relax-DTI

In order to estimate the unknown parameters of the signal represented by Eq. (5), we start by fitting the Relax-Kurtosis representation (4) to the diffusion-relaxation scattered MR signal using a non-linear least-squares cost function optimized via the Levenberg-Marquardt algorithm. In particular, we are interested in the unknown parameters of *R*(***ρ***|***θ***), i.e. T_1_, ${\text{T}}_{2}^{⋆}$, and PD. By using these values as initial estimates, the next step consists of fitting a diffusion part expressed by a diffusion tensor **D**. This is done by a non-linear least-squares method over the residuals defined between the data and a signal representation modified by the relaxation part

(11) $$r_{k}=S_{k}-R\left(\rho |\theta_{k}\right)\exp\left(W_{k}\gamma^{T}\right),$$

where *r*_*k*_ is the residual between the diffusion-weighted signal *S*_*k*_ from *k*-th acquisition direction over a fixed single *b*-value, $W_{k}=[-b_{k}u_{kx}^{2},-b_{k}u_{ky}^{2},-b_{k}u_{kz}^{2},-b_{k}u_{kx}u_{ky},-b_{k}u_{kx}u_{kz},-b_{k}u_{ky}u_{kz}]$ is the row from the design matrix for *k*-th acquisition, ***γ*** = [*D*_*xx*_, *D*_*yy*_, *D*_*zz*_, *D*_*xy*_, *D*_*xz*_, *D*_*yz*_] presents the elements of a second-order tensor, and *R*(***ρ θ***_*k*_) is the estimated relaxation contribution to the *k*-th sample. In short, we estimate three relaxation parameters (i.e. T_1_, ${\text{T}}_{2}^{⋆}$, and PD) and six elements of the diffusion tensor **D**.

#### Relax-SHORE

To fit the representation given by Eq. (9), we first fit the Relax-Kurtosis formula (4) using the nonlinear least-squares method solved by the Levenberg-Marquardt algorithm. Once the model is fitted to the signal, i.e. the set of parameters T_1_, ${\text{T}}_{2}^{⋆}$, PD, D and K are obtained, we calculate the relaxation part *R*(***ρ*** |***θ***) and multiply it with the 3D-SHORE basis

(12) $${\text{Φ}}_{nlm}^{\prime }(q)=R(\rho |\theta )G_{nl}(q)Y_{l}^{m}(u)$$

where Φ′_*nlm*_(**q**) we call the modified 3D-SHORE basis. Next, we estimate the coefficients of the bases (12) via the ordinary least-squares method ${\hat{a}}^{\prime }={\left(\Phi^{\prime T}\Phi^{\prime }\right)}^{-1}\Phi^{\prime T}S$ with ${\hat{a}}^{\prime }$ being the vector of estimated 3D-SHORE coefficients, $\hat{a_{nlm}^{\prime }},\Phi^{\prime }\in {\mathbb{R}}^{N\times M}$ is a design matrix with each column presenting the modified basis function Φ′_*nlm*_(**q**) with *M* being the number of basis atoms (e.g. for the radial order of *L* = 4/6 the number of functions is 22/50) and **S** ∈ ℝ^*N*^ is the vector of data samples used to fit the representation. Finally, the signal is reconstructed via the estimated coefficients $\hat{a_{nlm}^{\prime }}$ and we optimize the relaxation part via the Levenberg-Marquardt algorithm. In comparison to the initial step, this time, we optimize only the relaxation component, i.e., we look for the parameters T_1_, ${\text{T}}_{2}^{⋆}$ and PD.

Algorithm 1 **Relax-SHORE fitting procedure**

**Input:** Initial parameters: T_1_, ${\text{T}}_{2}^{⋆}$, PD, D, K.

**Output:** T_1_, ${\text{T}}_{2}^{⋆}$, PD, SHORE coefficients *a*′_*nlm*_.

**Variables:** *j* ← 1

**1** Fit the Relax-Kurtosis (4) to the scattered data via the Levenberg-Marquardt algorithm. Output: PD_0_, T_10_, ${\text{T}}_{20}^{⋆}$, *D*_0_, *K*_0_

**2** Set **x**_0_ with estimated parameters and **0** vector for SHORE coefficients: ${\text{x}}_{0}\leftarrow \left[{\text{PD}}_{0},{\text{T}}_{10},{\text{T}}_{20}^{⋆},0\right]$

**3 do**

**4** Calculate *R*(***ρ***|***θ***) part with T_1*j*_, ${\text{T}}_{2j}^{⋆}$, PD_*j*_.

**5** Modify the SHORE basis via Eq. (12). Output: **Φ**′*j*,

**6** Estimate the coefficients of the modified SHORE basis: ${\hat{a}}_{j}^{\prime }={\left(\Phi_{j}^{\prime T}\Phi_{j}^{\prime }\right)}^{-1}\Phi_{j}^{\prime T}S.$.

**7** Calculate the diffusion part with estimated modified SHORE coefficients ${\hat{a}}_{j}^{\prime }$

**8** Optimize the relaxation part *R*(***ρ***|***θ***). Output: ${\text{PD}}_{j+1},{\text{T}}_{1j+1},{\text{T}}_{2j+1}^{⋆}$

**9** $x_{j}\leftarrow \left[{\text{PD}}_{j},{\text{T}}_{1j},{\text{T}}_{2j}^{⋆},a_{j}^{\prime }\right]$

**10** *j* ← *j* + 1

**11 while** $|\frac{x_{j}-x_{j-1}}{x_{j-1}}|{|}_{\infty }>\epsilon$

This iterative algorithm is repeated unless the maximum norm of relative changes (MNRC) in estimated representation parameters between two consecutive iterations is smaller than or equal to *ϵ*, fixed in advance, i.e.

(13) $${\text{MNRC}}_{j}=||\frac{{\text{x}}_{j}-{\text{x}}_{j-1}}{{\text{x}}_{j-1}}|{|}_{\infty }\leq \epsilon$$

with $x_{j}=\left[{\text{PD}}_{j},{\text{T}}_{1j},{\text{T}}_{2j}^{⋆},a_{j}^{\prime }\right]$ being the parameter vector estimated in *j* − th iteration, the division between two vector parameters is carried out on a point-by-point basis and || · ||_∞_ is the maximum norm of the vector.

The optimization scheme presented here is compactly summarized in Algorithm 1. Note that the procedure is flexible as it enables to use other functional bases (Özarslan et al., 2013a; Ning et al., 2015), or even regularized-based solutions (Fick et al., 2016; Koch et al., 2019; Varela-Mattatall et al., 2020).

Once estimated the Relax-SHORE signal representation, we obtain PD, T_1_ and ${\text{T}}_{\text{2}}^{⋆}$ relaxation times and compute the generalized fractional anisotropy (GFA) from the coefficients of the ODF (Tuch, 2004), as well as the EAP-related indices including RTOP, RTAP, RTPP and mean squared displacement (MSD), all computed analytically from the estimated basis coefficients $\hat{a_{nlm}^{\prime }}$.

## 3 Materials and methods

### 3.1 In vivo diffusion-relaxation scattered MR data

We use the diffusion-relaxation scattered MR datasets from the MICCAI 2019 MUltidimensional DIffusion (MUDI) challenge (Pizzolato et al., 2020) that includes acquisitions from five healthy volunteers (2F, 3M, 19-46 years old). The subjects were scanned with a clinical 3T Philips Achieva scanner (Best, Netherlands) equipped with a 32-channel head coil. The single-shot PGSE EPI sequence was used with ZEBRA modifications as indicated by Hutter et al. (2018). The acquisition incorporated the following parameters: 28 inversion times (*TI* ∈ [20, 7322] ms), three echo times (*TE* ∈ {80, 105, 130 ms}), four b-value diffusion shells (*b* ∈ {500, 1000, 2000, 3000} s/mm^2^) and 105 different gradient directions leading to 1344 volumes in total. Other acquisition parameters were as follows: *TR* = 7500 ms, resolution = 2.5 × 2.5 × 2.5 mm^3^, pulse separation time/diffusion gradients length Δ/*δ* = 39.1/24.2 ms, FOV = 220 × 230 × 140 mm, parallel accelerated reconstruction via SENSE = 1.9, halfscan = 0.7, and multiband factor 2. Total scan time for a single subject was about 52 minutes.

### 3.2 In silico diffusion-relaxation scattered MR data

The ball and stick model (Behrens et al., 2003) was used to represent the diffusion part of the signal. Two sticks contribute each with the 35% of the signal, both with axial diffusivity *λ*_∥_ = 1.5 × 10^−3^ mm^2^/s and crossing at different angles are fixed, i.e. {90°, 75°, 60°, 45°, 30°, 15°}. The remaining 30% of the signal, is modelled as free water diffusion with diffusivity set to 1.5 × 10^−3^ mm^2^/s. The relaxation part is modelled using Eq. (3). The distribution of TIs, TEs, b-values and gradient directions were retrieved from the *in vivo* diffusion-relaxation scattered MR data. Other simulation parameters were as follows: PD = 100, T_1_ in the range [400, 3600] ms with a step size of 200 ms, ${\text{T}}_{\text{2}}^{⋆}$ in [10, 110] ms with a step size of 20 ms and IE = 2. Rician noise (Gudbjartsson and Patz, 1995) with the signal-to-noise ratio SNR ∈ [10, 30], defined in terms of the baseline acquisition (*b* = 0), was added to the ground-truth data.

### 3.3 Experimental setup

The Relax-SHORE method is compared with Relax-ADC (2), Relax-Kurtosis (4) and Relax-DTI (5). The initial parameters used for the non-directional methods are as follows: PD = PD_max_ with PD_max_ being the maximum value of the signal over all acquisitions, T_1_ = 800 ms, ${\text{T}}_{2}^{⋆}=60\;\text{ms}$, ADC = 10^−3^ mm^2^/s, and in the case of Relax-Kurtosis – D = 10^−3^ mm^2^/s and K = 0.5. These initial values are adopted for all voxels over the *in vivo* brain data. For synthetic experiments we fixed the initial PD parameter to be PD = 100. For all experiments, the radial order for Relax-SHORE is set to *L* = 4 unless otherwise specified and the scale *ζ* in Eq. (8) is computed using the formula *ζ* = 1/(8*π*^2^*τ* MD) where MD is the mean diffusivity retrieved from the diffusion tensor with *τ* = −Δ *δ/*3 being the effective diffusion time.

## 4 Results

This section presents the numerical evaluations of diffusion-relaxation scattered MR signal representation fitting methods using both *in silico* and *in vivo* data.

### 4.1 In silico experiments

**Figure 1:**
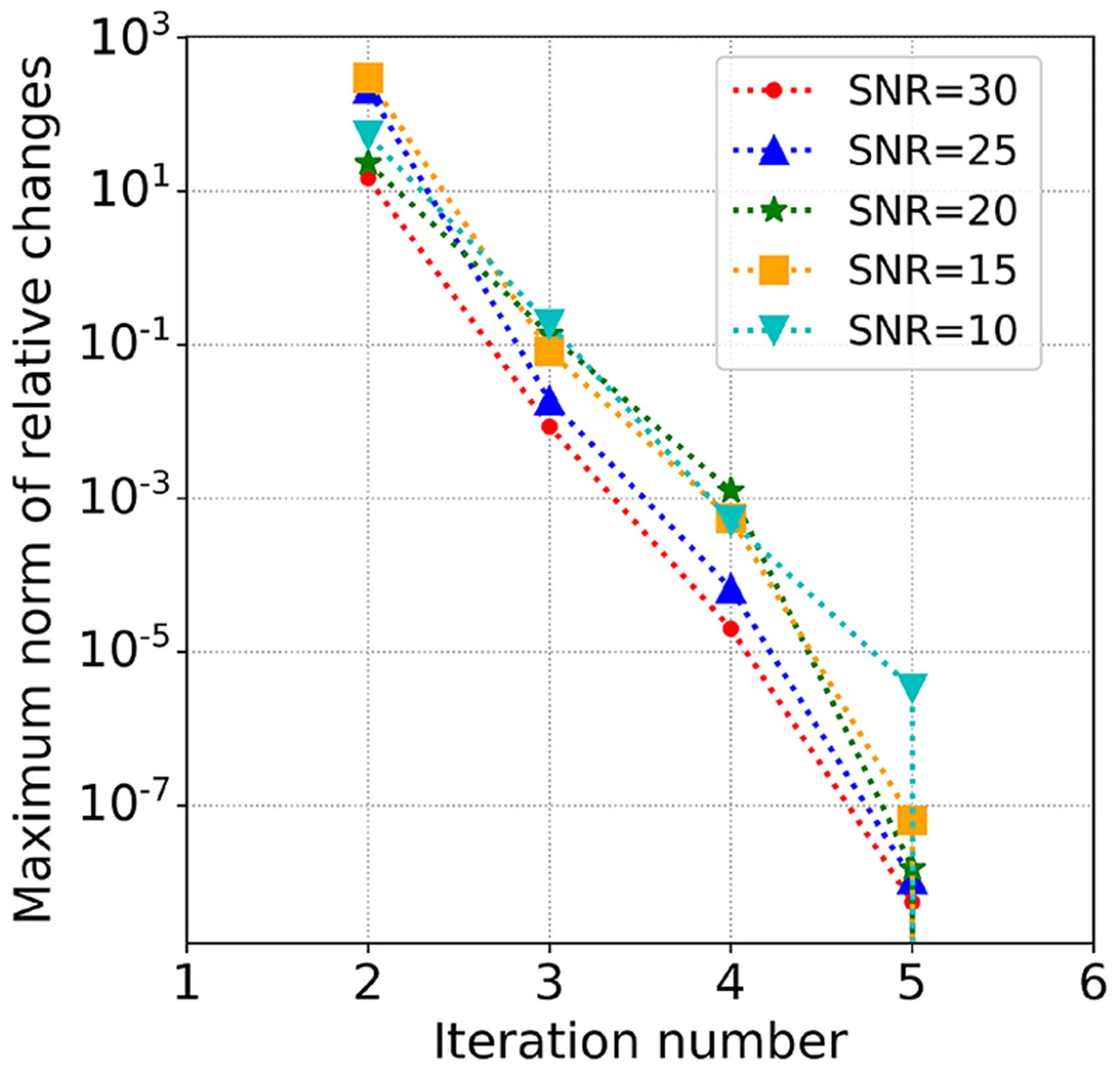
The maximum norm of relative changes of the Relax-SHORE parameters in function of iteration number under different SNRs. The Relax-SHORE model was fitted to synthetically generated data using the ball & stick model with different ground truth parameters including T_1_ ∈ [400, 3600] ms with a step size of 200 ms, ${\text{T}}_{2}^{⋆}\in [10,110]$ with a step size of 20 ms and angles defined between two sticks 90°, 75°, 60°, 45°, 30° and 15°.

We first check the convergence of the iterative fitting process in the Relax-SHORE algorithm under varying signal-to-noise ratios (SNRs) and with various acquisition parameters (i.e. *TE, TI*, and *b*-value). In this experiment, we fit the signal representation (9) using all 1344 volumes and evaluate the changes in the Relax-SHORE representation, i.e. the changes in the vector **x** in function of iteration number. We varied the SNR (i.e. SNR ∈ {10, 15, 20, 25, 30}) and calculated the maximum norm of the relative changes of model parameters with Eq. (13) including all 1344 volumes. These maximum norms were averaged over all noise instances within a specific SNR and relaxation times (i.e. T_1_ in the range of [400, 3600] ms with a step size of 200 ms, ${\text{T}}_{2}^{⋆}$ belonged to the interval of [10, 110] with a step size of 20 ms) and angles defined between two sticks in the ball & stick model included 90°, 75°, 60°, 45°, 30° and 15°. The results of this experiment are shown in Fig. 1. The maximum norm of relative changes of the representation vector **x** decreases with the iteration number, which means that the solution converges. With only five iterations, the Relax-SHORE method was able to reach a maximum norm of relative changes below 10^−5^ with all evaluated SNR levels.

**Figure 2:**
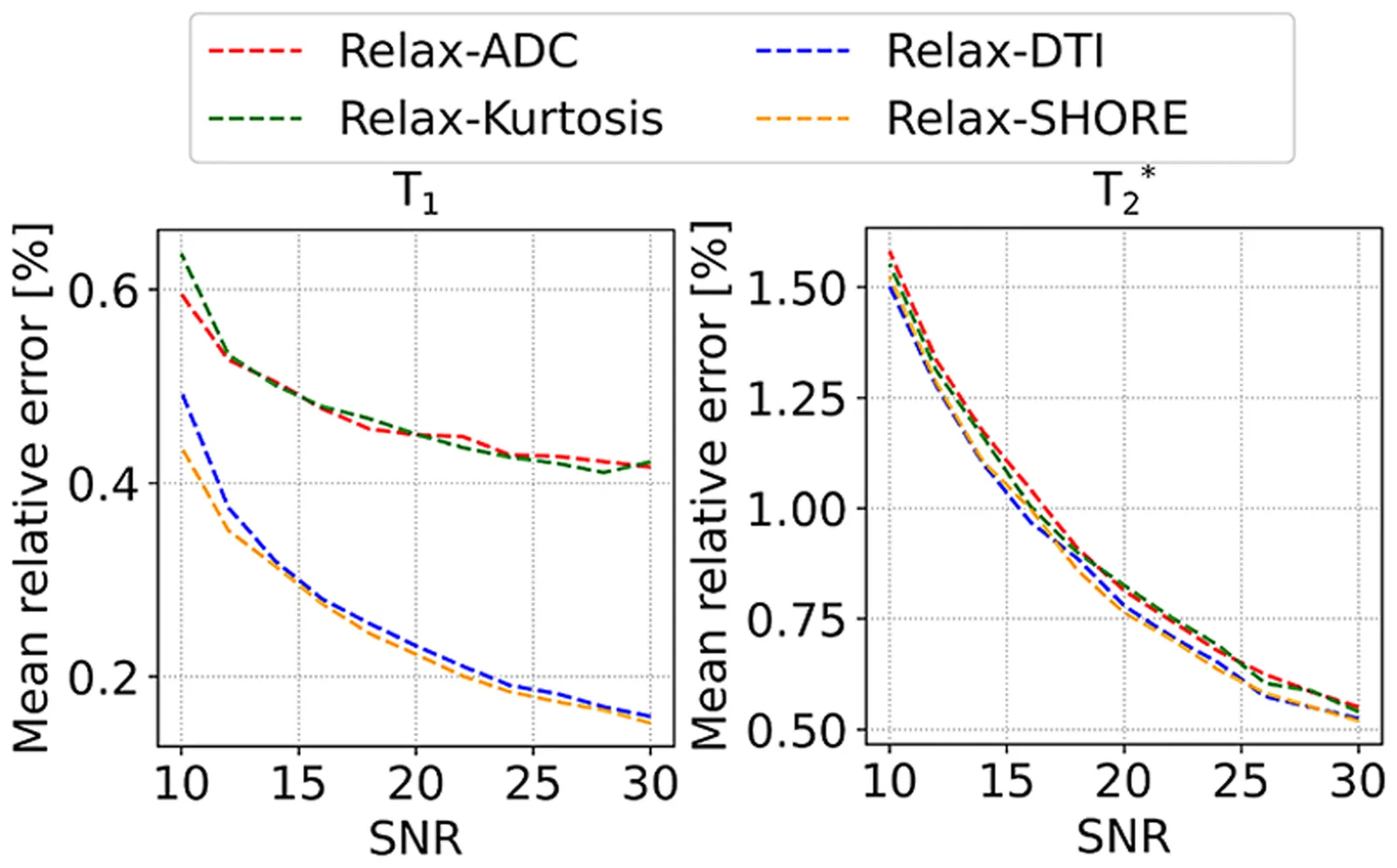
Mean relative error (MRE) of T_1_ and ${\text{T}}_{2}^{⋆}$ estimations computed for different diffusion-relaxation scattered MR signal representations (i.e. Relax-ADC, Relax-Kurtosis, Relax-DTI, and Relax-SHORE) in function of SNR. The MRE was calculated between the ground-truth values and the estimated ones from the noisy data at a specified SNR, and aggregated over all combinations of T_1_, ${\text{T}}_{2}^{⋆}$ and angles given between two sticks.

**Figure 3:**
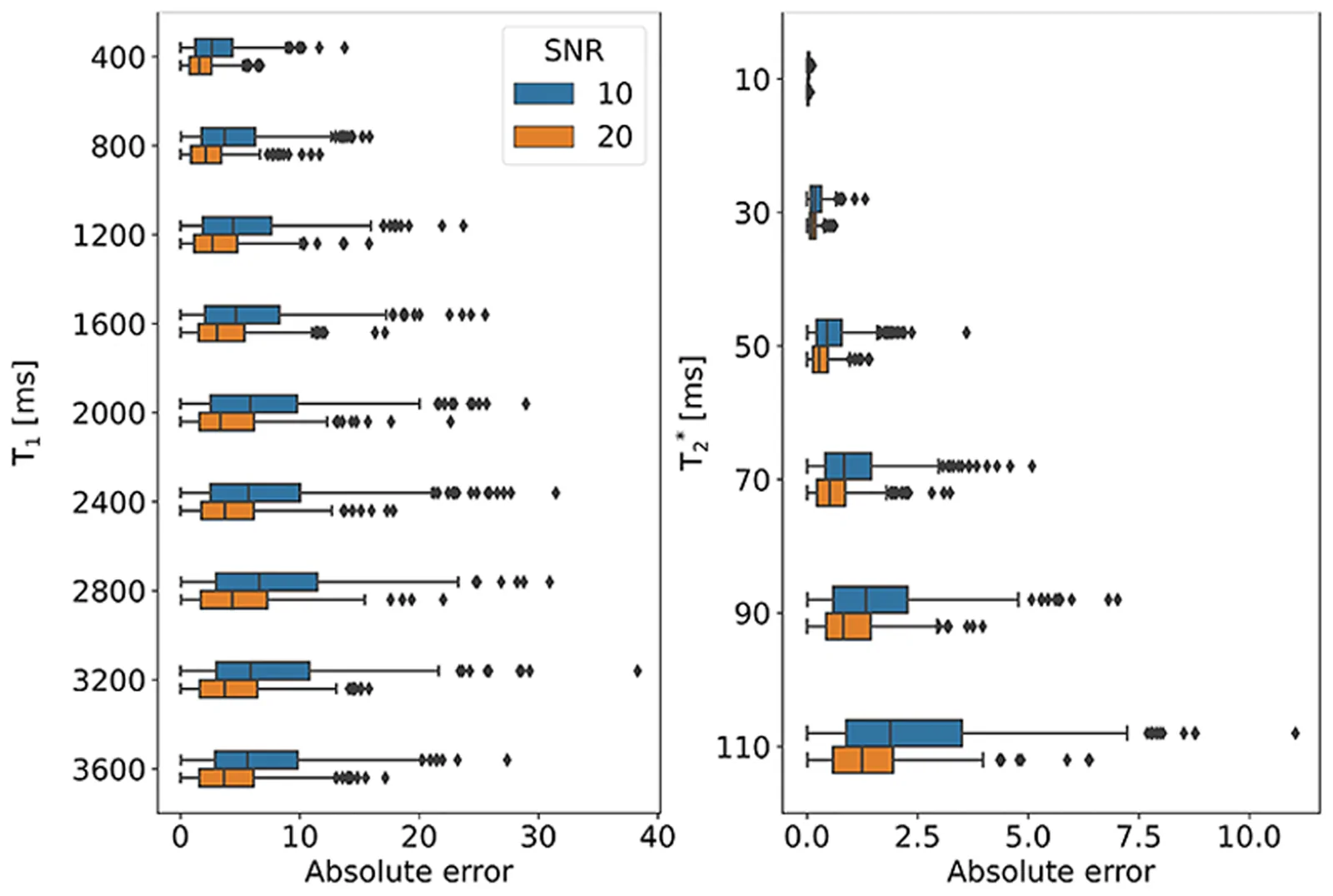
Absolute errors of T_1_ and ${\text{T}}_{2}^{⋆}$ estimation with Relax-SHORE and under different experimental T_1_ and ${\text{T}}_{2}^{⋆}$ values, all evaluated under SNR ∈ {10, 20}. The box plots present the quartiles (i.e. first, second and third), while the whiskers show the minimal and maximal absolute errors.

We present now two experiments on the robustness of T_1_ and ${\text{T}}_{2}^{⋆}$ estimation from diffusion-relaxation scattered MR data as a function of the SNR. In the first experiment, we compared the four methods mentioned before, i.e. Relax-ADC, Relax-Kurtosis, Relax-DTI, and Relax-SHORE, and evaluated the mean relative error (MRE) calculated between the ground-truth relaxation values and the estimated ones from the noisy data at a specified SNR. The MRE is reported in %. Again, the error is averaged including all combinations of simulated T_1_, ${\text{T}}_{2}^{⋆}$ and angles defined between two sticks. The results of this experiment are illustrated in Fig. 2. In general, the estimation error decreases with increasing the SNR. For the Relax-SHORE, the T_1_ estimation error is the lowest among other tested methods used to retrieve the relaxation parameters, while in the case of ${\text{T}}_{2}^{⋆}$, we observe a comparable MRE for all tested techniques. In a second experiment, for which results are illustrated in Fig. 3, we report the distribution of absolute errors of T_1_ and ${\text{T}}_{2}^{⋆}$ estimates using Relax-SHORE from diffusion-relaxation scattered MR data under two fixed SNR, i.e. SNR ∈ {10, 20}. The configuration used to generate the synthetic data for this experiment strictly follows the scheme used previously. Contrary to the previous experiment, this one focuses on the absolute differences between the original and the estimated relaxation times from the noisy data at a given SNR. In the figure we present the first, second (median), and third quartiles of the absolute error and whiskers showing the minimal and maximal absolute errors. Note that the absolute error also depends on the simulated relaxation times, i.e. the median and the variability of the error increase for higher relaxation times.

**Figure 4:**
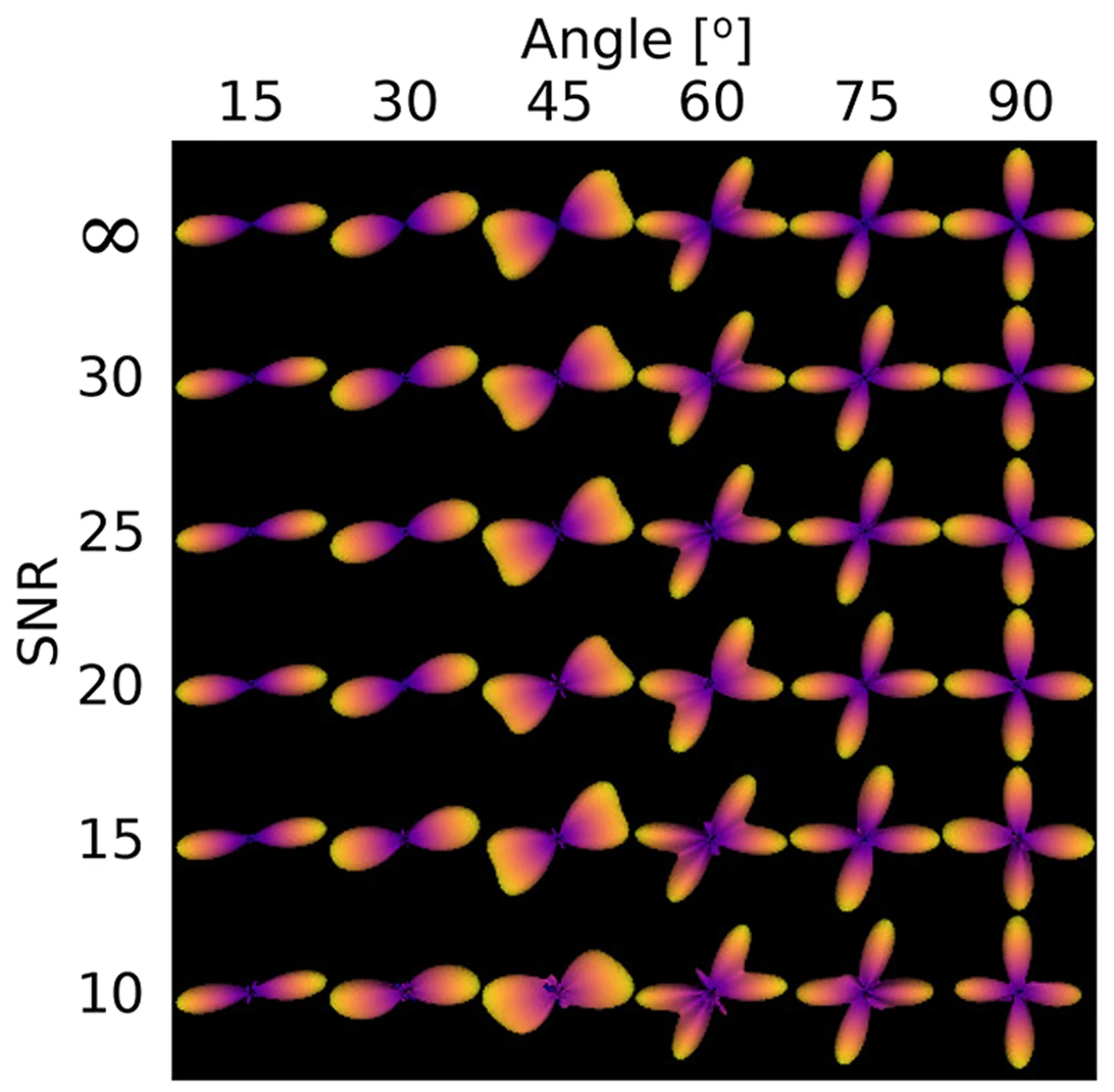
The ODFs computed from the estimated Relax-SHORE representation of diffusion-relaxation scattered MR synthetic signal under different SNRs and crossing angles fixed between two sticks.

We now analyze the accuracy of the Relax-SHORE in describing the directional information of the diffusion process from diffusion-relaxation scattered MR data. Specifically, we analyze the capabilities to distinguish the main fibre direction in a two-sticks crossing scenario. To this end, we generate the diffusion-relaxation scattered MR signals with longitudinal relaxation *T*_1_ = 800 ms and transverse relaxation ${\text{T}}_{2}^{⋆}=90$ ms combining different SNRs and angles given between two fibres, and estimate the ODFs using the Relax-SHORE representation with radial order *L* = 6. In this experiment, the ODFs were estimated for 11554 equally spaced points on a unit sphere generated with the repulsion algorithm using Diffusion Imaging in Python (DIPY) package (Jones et al., 1999; Garyfallidis et al., 2014). The results presented in Fig. 4 show that two fibres can be distinguished once the angle between them is defined to be at least 45°. For smaller angles, the method determines only one single direction representing two fibres. Note the technique exhibits robustness regarding the SNR level of the baseline signal (*b* = 0), producing only slightly deteriorated results at SNR = 20, which is a typical value in diffusion MRI acquisitions.

### 4.2 In vivo experiments

**Figure 5:**
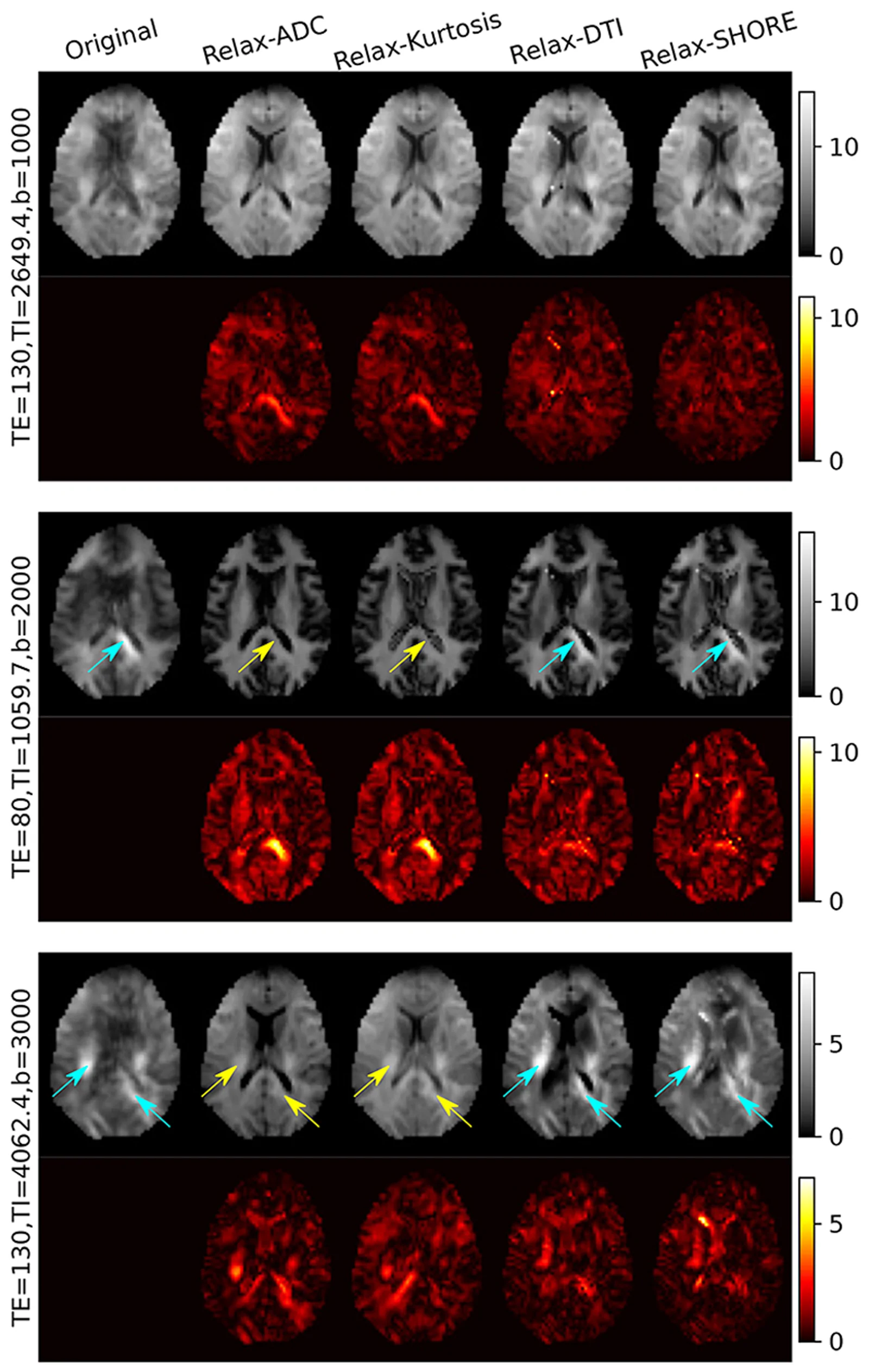
The predictions (top rows) and absolute errors (bottom rows) between the predicted signal from unseen acquisition and the original *in vivo* MR data. The fitting procedure for each method is based on the 536 chosen volumes. The absolute errors are presented between a prediction and original data from a selected volume at a specified configuration given in *T E* [ms], *T I* [ms] and *b*-value [s/mm^2^]. The arrows show the superior corona radiata and splenium of the corpus callosum where the signal has been diminished by Relax-ADC and Relax-Kurtosis methods (marked in yellow) while preserved with Relax-DTI and Relax-SHORE (cyan arrows).

We now present the results for *in vivo* diffusion-relaxation scattered MR data acquired from healthy subjects (see section 3.1). We start with a prediction experiment that aims at signal reconstruction from a selected subject (cdmri0011) using the four methods mentioned above, namely Relax-ADC, Relax-Kurtosis, Relax-DTI and Relax-SHORE. The model was fitted to the 536 selected samples, while the prediction was made for the remaining 808 unseen samples (1344 acquisitions in total). The subsampling scheme considers all *b*-values, reducing also the amount of information in terms of *TE* and *TI*. To subsample the data, we choose the volumes with the highest coefficient of variation (CV)

(14) $${\text{CV}}_{i}=\frac{\sigma_{i}}{\mu_{i}},\;i=1,\ldots ,1344$$

with CV_*i*_ being the coefficient of variation calculated for *i*-th volume out of 1344, *µ*_*i*_ and *σ*_*i*_ are the sample mean and sample standard deviation of *i*-th volume in the fore-ground area. For each volume characterized by a different acquisition parameter set we computed the mean and the standard deviation of the data within the whole brain area. That allowed to compute the CV_*i*_ of each volume. Then, we sorted the CV_*i*_ decreasingly and chose the first 536 corresponding acquisition parameter sets. The experiment is illustrated in Fig. 5, where the predicted axial slices are compared to the actual ones at *b* = 1000, 2000 and 3000 s/mm^2^. We observe both the Relax-ADC and Relax-Kurtosis provide significantly increased errors compared to Relax-DTI and Relax-SHORE. The regions of the superior corona radiata and the splenium of the corpus callosum (see yellow arrows in Fig. 5) have been underestimated at *b* = 2000 and *b* = 3000 s/mm^2^ with the non-directional diffusion representations, i.e. Relax-ADC and Relax-Kurtosis, while they are recovered with Relax-

In the next experiment, we quantify the diffusion-relaxation scattered MR signal prediction over the white matter (WM), gray matter (GM), and cerebral spinal fluid (CSF) areas. We predict the signal using the same configuration setup as used in the previous experiment and calculate the average MSE over all five subjects. To calculate the prediction MSE over the WM/GM/CSF, we register the fractional anisotropy, retrieved from the DTI at *b* = 1000 s/mm^2^, to the standard space using a non-linear transformation preceded by a linear initialization with seven degrees-of-freedom, correlation ratio cost function and spline-based interpolation using the FSL (Analysis Group, FMRIB, Oxford, UK.; Smith et al. (2004)) and retrieve WM/GM/CSF labels from the MNI152 standard-space T1-weighted average structural template (Grabner et al., 2006). The results of this experiment are included in Table 1. We observe the proposed Relax-SHORE method exhibits the lowest error amongst all tested methods within the WM region across all three configurations while still being comparable in the GM region. This suggests that Relax-SHORE can correctly reproduce complex neuroarchitecture leading to significantly lower average MSE in comparison with the methods representing the diffusion using scalar values such as the Relax-ADC or Relax-Kurtosis. In CSF, the Relax-SHORE gives comparable or slightly worse results than other solutions.

**Figure 6:**
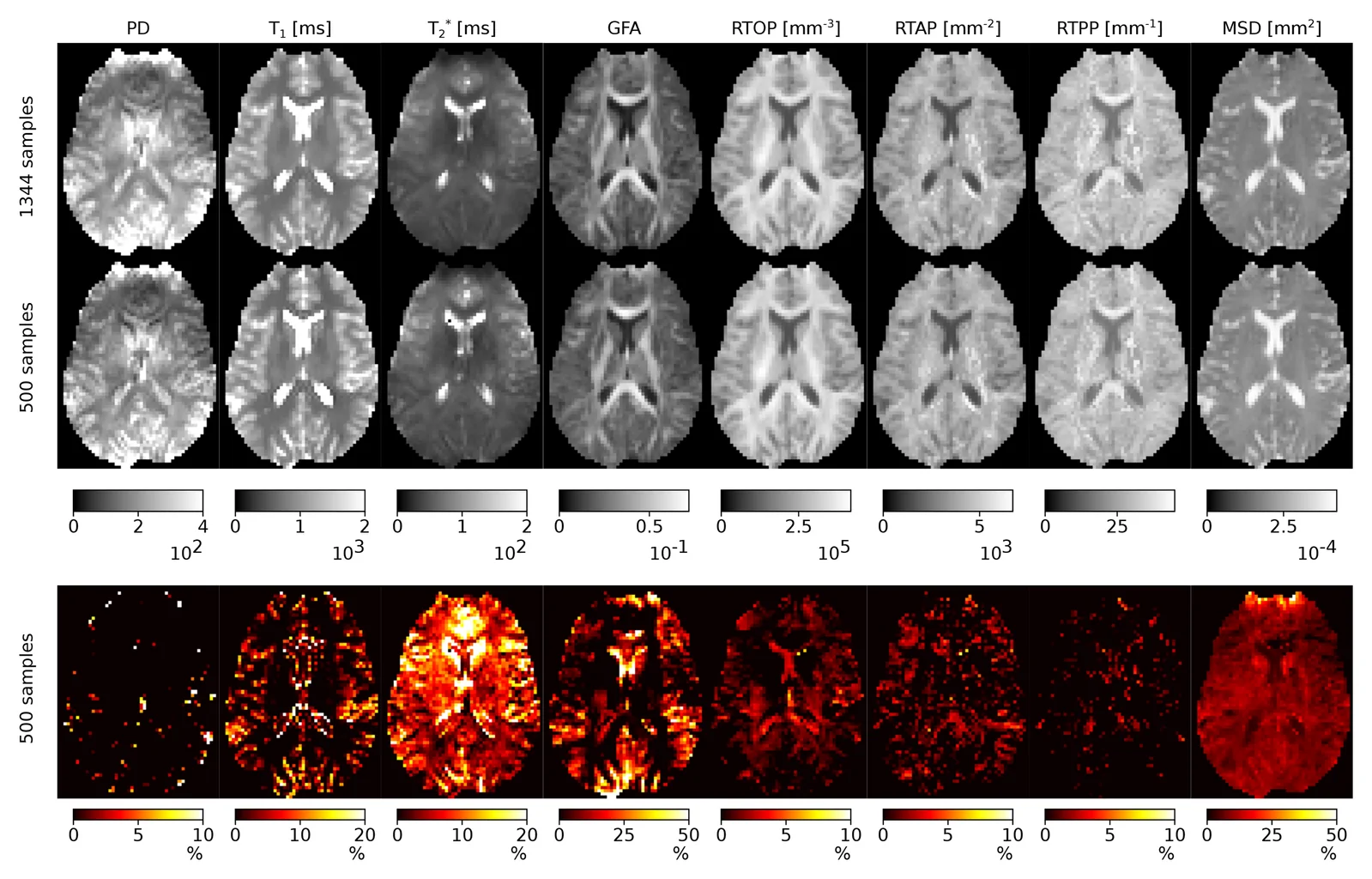
Relaxation and diffusion measures (PD, T_1_, T_2_^⋆^, GFA, RTOP, RTAP, RTPP, MSD) derived from *in vivo* diffusion-relaxation scattered MR data using Relax-SHORE method (top). Each measure is computed in a fully-sampled case using all available information (i.e. 1344 samples) and in a reduced scenario only considering 500 samples, both cases including different echo times, inversion times and diffusion acquisition parameters (b-values and gradient directions). The bottom row reports the relative error of the measures retrieved from 500 samples in comparison to the fully-sampled case. Mean relative errors of the measures over the brain area estimated from subsampled data: PD 4.15%, T_1_ 4.29%, ${\text{T}}_{2}^{⋆}$ 7.37%, GFA 12.45%, RTOP 1.14%, RTAP 1.38%, RTPP 1.12%, MSD 8.41%.

In the next experiment, we estimate relaxation and diffusion scalar maps using the Relax-SHORE approach with fully-sampled data and a subsampled case. The quantitative maps were estimated both considering all the available samples (1344) and a subsampled scenario (i.e. 500 volumes). We selected all available non-diffusion volumes (i.e. *b* = 0, while in the case of diffusion-weighted data, we selected 28 (*b* = 500 s/mm^2^), 84 (*b* = 1000 s/mm^2^), 138 (*b* = 2000 s/mm^2^) and 166 volumes (*b* = 3000 s/mm^2^), every shell covering all available TI and TE values. Three relaxation maps, i.e. PD, T_1_, ${\text{T}}_{2}^{⋆}$, and five diffusion indices including the GFA and EAP-based measures (i.e. RTOP, RTAP, RTPP, MSD) were retrieved in both scenarios (i.e. 1344 and 500 volumes) and depicted together in Fig. 6 along with the relative errors computed for the subsampled case. The relative errors of the measures retrieved from 500 samples were calculated with respect to the fully-sampled case (i.e. 1344 volumes) and multiplied by 100%. Note that the EAP-based indices were retrieved from the signal representation without any regularization (see Fick et al. (2016); Koch et al. (2019); Varela-Mattatall et al. (2020) for some regularization methods).

In the following two *in vivo* experiments, we evaluate the accuracy of Relax-SHORE with respect to the anisotropy of diffusion and the robustness of the method in the case of a reduced number of samples used to fit the representation.

**Figure 7:**
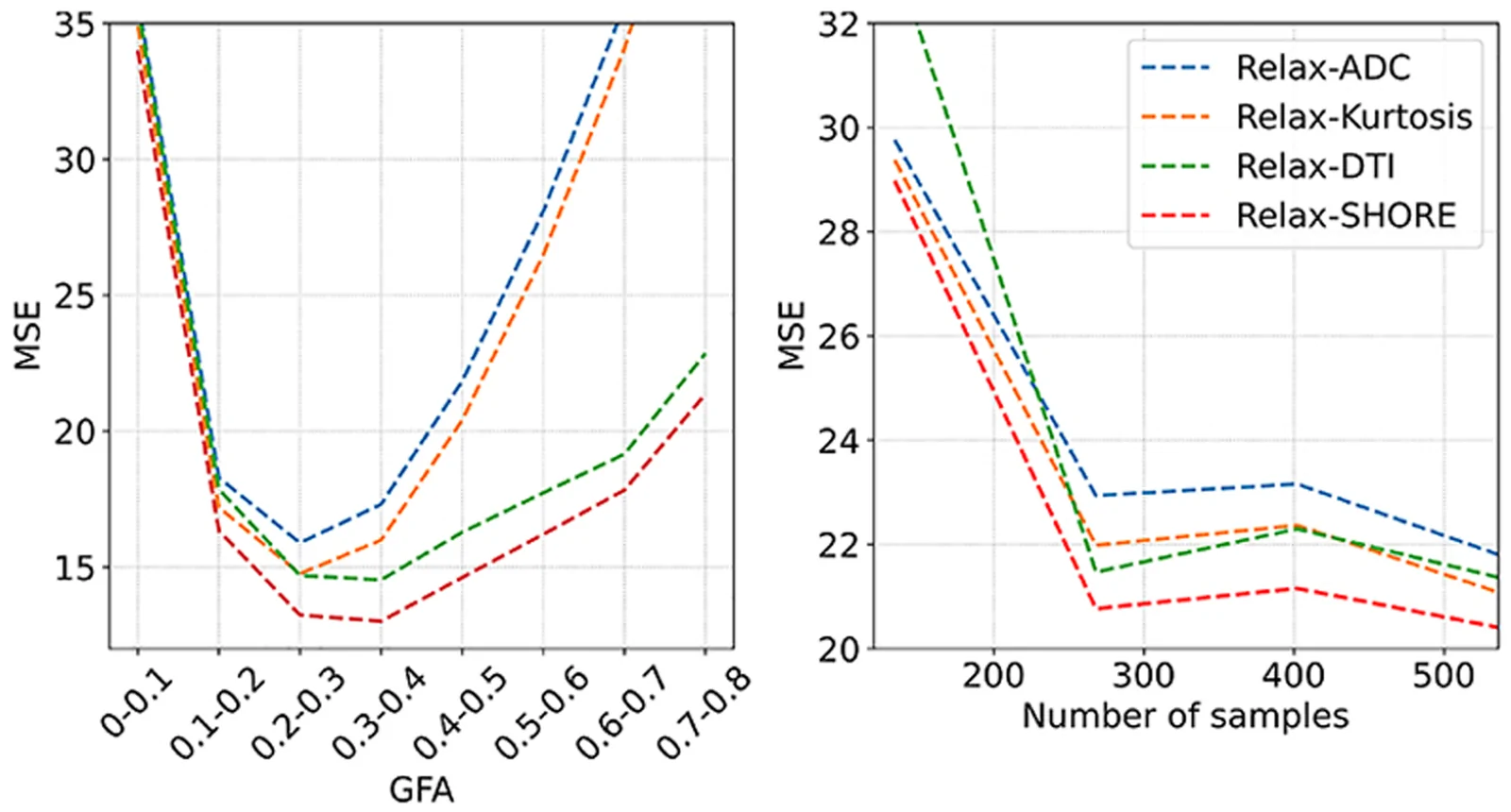
The MSE between the original *in vivo* diffusion-relaxation scattered MR signal and a prediction applying four representations, namely the Relax-ADC, Relax-Kurtosis, Relax-DTI, and Relax-SHORE. The left figure presents the MSE of a predicted signal as a function of the anisotropy level of diffusion process revealed using the GFA measure, while the right one shows the MSE in terms of the number of samples used in the estimation procedure. For the experiment evaluating the MSE as a function of the GFA measure, all 1344 volumes are used to fit representations, while in the case of the subsampling experiment all four methods consistently use a reduced number of samples.

The results of the first experiment are illustrated in Fig. 7(left), where we compare the MSE of the signal representation estimated by the Relax-SHORE with Relax-ADC, Relax-Kurtosis, and Relax-DTI as a function of the GFA. We divided the brain into eight clusters, each characterized by a different range of anisotropy of diffusion as a function of GFA. For each cluster, we calculated the MSE between the original data and the fitted representation. The results show that Relax-SHORE outperforms all other methods when GFA > 0.1.

The next experiment evaluates the robustness of Relax-SHORE under a limited number of samples used to fit the representation. We fit the representation using a decreasing number of samples and calculate the MSE between the prediction for unseen acquisition and original data.

In this experiment, we employ the subsampling scheme following the Eq. (14). For each subsampling level, the volumes characterized by the highest CV are used in the estimation process. The results of this experiment are presented in Fig. 7(right). For all of the tested models, the highest MSE is obtained with the lowest number of samples as expected. Overall, the Relax-SHORE is characterized by the lowest MSE. We recall that the Relax-SHORE method needs 25 (3+22) parameters (relaxation+diffusion) for radial order of *L* = 4 to be estimated in comparison to 4 (3+1) and 5 (3+2) coefficients required by the Relax-ADC and Relax-Kurtosis, respectively.

In the last *in vivo* experiment, we fit Relax-ADC, Relax-Kurtosis, and Relax-SHORE to the original diffusion-relaxation scattered MR data using all 1344 volumes. Then, we calculate the normalized MSE (NMSE) between the original signal and fitted representations and check how the NMSE changes across unique parameters used to acquire the data (i.e. *TI, TE*, and *b*).

**Figure 8:**
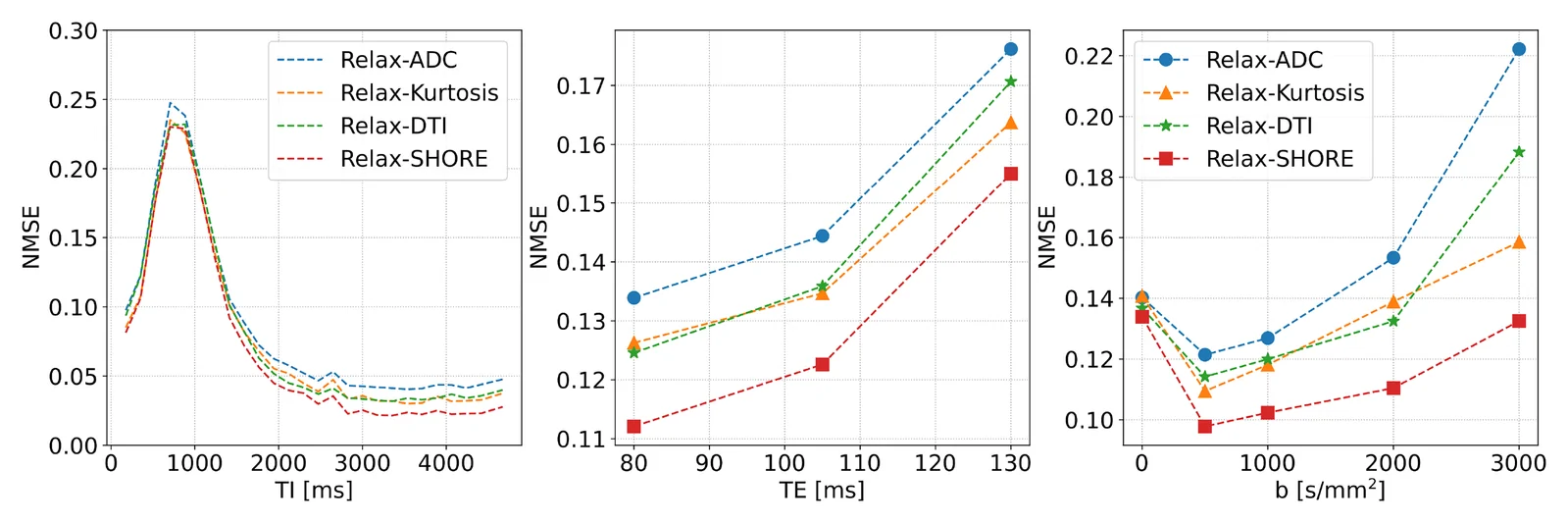
The normalized mean square error (NMSE) between the original *in vivo* diffusion-relaxation scattered MR signal and fitted representations under selected acquisition parameters: *TI* (left), *TE* (middle) and *b*-value (right). In all cases, the fitting procedure is applied to all 1344 volumes.

In Fig. 8, we show three plots presenting the NMSE between fitted representations and the volumes characterized by different *TI, TE*, and *b*-values, respectively. Notice the NMSE at a single *TI* uses all available information throughout *TE* and *b*-value to calculate the error, the NMSE at a single *TE* uses all available volumes acquired at different *TI* and *b*-values, and eventually the NMSE at a single *b*-value – all volumes acquired at different *TE* and *TI*.

All the methods manifest the lowest NMSE above *TI* = 2000 ms with a peak value visible around *TI* = 800 ms that is related to the inversion of the signal. Next, in Fig. 8(middle), we present the NMSE of a predicted signal as a function of a varying *TE* parameter. Again, the NMSE is calculated at a fixed *TE* using all information available throughout *TI* and *b*-value. Here, the NMSE of all four methods increases consistently with *TE*, and the Relax-SHORE is discriminated with the lowest error among the three other techniques. In the last experiment (see Fig. 8(right)), we check how the NMSE of a reconstructed signal versus the original depends on the *b*-value. The Relax-SHORE technique reaches the lowest NMSE in contrast to other methods in all examined *b*-values. That result is expected since Relax-SHORE is intended to have a high fidelity to the data at all *b*-values.

## 5 Discussion

### 5.1 Synthetic results

An iterative estimation technique for Relax-SHORE has been introduced, which alternately optimizes the relaxation and diffusion parts of the signal. The analysis for synthetic diffusion-relaxation scattered MR data indicates that the procedure stabilizes quickly for a range of tested SNRs and acquisition parameters (Fig. 1). Note that the relative changes of the parameter values are small after just four iterations which means that acceptable results are achieved over a few iterations. Although we have no formal proof of the convergence, we did not observe a divergent behaviour of the optimization scheme.

Results presented in Fig. 2 indicate that introducing a full propagator based approach, like Relax-SHORE, provides the more precise estimation of T_1_ relaxation time. The absolute error in estimation of both relaxations depends on the SNR of the signal (see Fig. 3). The error and its variability do not change significantly with the change of the T_1_ value. However, we observe that ${\text{T}}_{2}^{⋆}$ estimation error and its variability increases with increasing the transverse relaxation time. It is an expected behaviour as in the case of higher transverse relaxations, the relative changes of the function $f\left(TE;{\text{T}}_{2}^{⋆}\right)=\exp\left(-\frac{TE}{{\text{T}}_{2}^{⋆}}\right)$ between two ${\text{T}}_{2}^{⋆}$ values are rather small. Therefore the difference in solutions for long ${\text{T}}_{2}^{⋆}$ times (e.g. 100 and 110 ms) produces relative low changes in *f*, while the same absolute difference in two short ${\text{T}}_{2}^{⋆}$ relaxations (e.g. 20 and 30 ms) produces high *f* value relative change.

The proposed approach enables to estimate the ODFs from scattered acquisitions and under a relatively low SNR of the baseline signal, as shown in Fig. 4. However, in this study, we assumed only a two-crossing fibre scenario. Relax-SHORE enables to distinguish two fibres once the angle between them is defined to be at least around 45° which is consistent with the previous report on 3D-SHORE basis (Fick et al., 2015).

### 5.2 In vivo *data*

In general, the proposed Relax-SHORE method is characterized by a relatively low error in a signal prediction task compared to Relax-ADC, Relax-Kurtosis, and Relax-DTI methods over the WM area (see Table 1). The scalar Gaussian-like and non-Gaussian-like techniques (i.e. Relax-ADC, Relax-Kurtosis) introduce errors in highly anisotropic structures such as the superior corona radiata or the splenium of the corpus callosum (see the yellow arrows in Fig. 5). However, the error of the signal predicted with Relax-SHORE in CSF is typically amplified compared to that obtained with Relax-Kurtosis (Table 1). This behaviour is mainly due to the isotropic nature of the diffusion profile inside the CSF and a relatively low SNR compared to WM/GM regions. In CSF, Relax-SHORE is therefore an over-complicated model to represent the signal. All in all, Relax-SHORE can better characterize diffusion in WM/GM regions than the previous techniques when considering a vast range of *b*-values.

The added value of the Relax-SHORE is the ability to estimate the diffusion microstructural indices from the scattered diffusion-relaxation dataset.

However, one must bear in mind that introducing complex models characterized by a larger number of degrees of freedom might be more problematic in scattered acquisitions such as ZEBRA (Hutter et al., 2018), leading to potential instabilities in the estimation process. The experiment depicted in Fig. 7(right) confirms that with Relax-SHORE, though more coefficients are required to be estimated, we observe no increase in the MSE of a predicted signal for a low number of samples compared to scalar-based diffusion representations such as the Relax-ADC or Relax-Kurtosis.

We observe a consistent reduction in the MSE for all methods with the increase in the sample number used to predict the signal.

Finally, we discuss the effects of acquisition parameters (i.e. *TI, TE* and *b*-value) on the fitted signal representation. Concerning *TI*, one can notice a peak value in the NMSE depicted in Fig. 8(left) around *TI* = 800 ms that is consistently observed for all tested representations. This error is located in the correspondence of the signal inversion point. This effect has been recently mentioned by Pieciak et al. (2021) for DTI and MAP-MRI techniques, and in the case of biophysical models by Ciupek et al. (2021). About the *TE*, we can observe a decrease in the NMSE for shorter *TEs* that results from a higher SNR of the signal. The last of the three experiments discussed here is the functional dependence of the NMSE with the *b*-value. Relax-SHORE outperforms both Relax-ADC and Relax-Kurtosis, especially under a higher *b*-value regime. In the middle, the Relax-Kurtosis arises as it catches both Gaussian and non-Gaussian diffusion profiles (Jensen and Helpern, 2010), but still, two scalars are not enough to fully present the anisotropic nature of diffusion in the brain.

### 5.3 Study limitations and future directions

The multi-parametric sequences such as the ZEBRA (Hutter et al., 2018) introduce a new possibility to quickly acquire a large amount of diffusion-relaxation scattered MR data, significantly reducing the scan time at once. These versatile sequences open new opportunities and challenges in modelling combined information from relaxation and diffusion components. We proposed to modify the functional basis to include both the relaxation and diffusion parts and then iteratively optimize the signal representation. Although the presented solution can retrieve the quantitative EAP-related measures robustly, it uses an ordinary least-squares approach to fit the functional basis. One could extend the fitting procedure including a regularization term, as suggested in Fick et al. (2014); Koch et al. (2019), or apply various constraints on the propagator (Haije et al., 2020). Despite this, our goal behind the methodology presented here was not to take all these recent advances in propagator modelling into account but instead, to introduce a general approach to engage the relaxation and diffusion parts mutually. Note we used a non-linear least-squares fitting procedure to optimize the relaxation part but one could go a step further and investigate more advanced optimization techniques.

In the present work, we consider a single relaxation compartment. Consequently, the T_1_ and T_2_ values aggregate all information coming from different compartments. Such an assumption may be regarded as a limitation, as recent advances in biophysical modelling aim at the representation of diffusion and relaxation properties for each tissue compartment independently Jelescu et al. (2020); Topgaard (2020).

However, the multicompartment diffusion-relaxation spectra estimation requires a large number of measurements.

Common approaches of such detailed signal analysis are based on the predefined dictionaries designed for a discrete number of possible parameter values (Slator et al., 2021). The values are positioned into the kernel function that models diffusion-relaxation process. Each additional parameter significantly increases the complexity of the problem. For example the T_1_-T_2_^⋆^-relaxation dictionary comprising information about 50 different T_1_ and 50 different T_2_^⋆^ values is made of 2500 atoms. Adding a simple diffusion model based only on the apparent diffusion coefficient, i.e. *S*(*b*) = exp(−*bD*), adds another level of complexity and produces an even larger dictionary.

## 6 Conclusion

We have presented a compact signal representation method that can handle diffusion-relaxation scattered MR signals acquired under different echo times, inversion times, and *b*-values. The proposed Relax-SHORE method allows optimizing the relaxation and diffusion parts alternately in an iterative process thus enabling the representation of relaxation while guaranteeing the fidelity to the diffusion signal and the estimation of indices provided by long-established diffusion propagator methods. The proposal can better represent the properties of diffusion-relaxation scattered MR signal, including its directional nature, and model the quantitative characteristics of the brain tissue from considerably reduced acquisition schemes.

## Figures and Tables

**Table 1:** Averaged mean square error (MSE in [a.u.]) of diffusion-relaxation scattered MR signal prediction calculated over all five subjects including four models, namely Relax-ADC, Relax-Kurtosis, Relax-DTI and Relax-SHORE. The fitting procedure for each method employs 536 volumes. The MSE parameter is provided within the white matter (WM), gray matter (GM) and cerebrospinal fluid (CSF) areas for the acquisitions under different configuration setup specified by the echo time (*TE* in [ms]), inversion time (*TI* in [ms]) and *b-*value (*b* in [s/mm^2^]), and it is calculated between original and predicted data for unseen acquisitions.

	*TE* = 130, *TI* = 2649.4, *b* = 1000			*TE* = 80, *TI* = 1059.7, b = 2000			*TE* = 130, *TI* = 4062.4, *b* = 3000		
	WM	GM	CSF	WM	GM	CSF	WM	GM	CSF
Relax-ADC	3.299	3.554	2.909	4.080	4.098	3.224	1.531	0.910	1.614
Relax-Kurtosis	2.749	**3.100**	**2.425**	4.158	4.123	2.796	1.326	0.945	**0.812**
Relax-DTI	2.801	3.592	3.307	2.870	3.654	2.999	0.799	**0.783**	1.513
Relax-SHORE	**2.352**	3.212	2.907	**2.601**	**3.551**	**2.719**	**0.707**	0.811	1.695
